# Transition from pediatric to adult care in patients with Turner syndrome in Italy: a consensus statement by the TRAMITI project

**DOI:** 10.1007/s40618-024-02315-4

**Published:** 2024-02-20

**Authors:** T. Aversa, L. De Sanctis, M. F. Faienza, A. Gambineri, A. Balducci, R. D’Aprile, C. Di Somma, C. Giavoli, A. Grossi, M. C. Meriggiola, E. Profka, M. Salerno, S. Stagi, E. Scarano, M. C. Zatelli, M. Wasniewska

**Affiliations:** 1 Department of Human Pathology of Adulthood and Childhood, University of Messina, Messina, Italy; 2 Pediatric Unit, University Hospital “G. Martino”, Via Consolare Valeria N. 1, 98124 Messina, Italy; 3 Pediatric Endocrinology, Regina Margherita Children Hospital, Turin, Italy; 4 Department of Public Health and Pediatric Sciences, University of Turin, Turin, Italy; 5 Department of Precision and Regenerative Medicine and Ionian Area, University of Bari “Aldo Moro”, 70124 Bari, Italy; 6 Division of Endocrinology and Diabetes Prevention and Care, Department of Medical and Surgical Sciences (DIMEC), IRCCS Azienda Ospedaliero – Universitaria di Bologna, Bologna, Italy; 7 Pediatric Cardiology and Adult Congenital Heart Disease Program, Department of Cardio - Thoracic and Vascular Medicine, IRCCS Azienda Ospedaliero - Universitaria Di Bologna, Bologna, Italy; 8 Department of Women’s and Children’s Health, University of Padua, Padua, Italy; 9 A.Fa.D.O.C. Association OdV, Vicenza, Italy; 10 Unit of Endocrinology, AOU Federico II, Naples, Italy; 11 Endocrinology Unit, Fondazione IRCCS Cà Granda Ospedale Maggiore Policlinico, Milan, Italy; 12 Endocrine Pathology of Chronic and Post-Tumor Diseases Unit, “Bambino Gesù” Pediatric Hospital, Rome, Italy; 13 Division of Gynecology and Physiopathology of Reproduction, Department of Medical and Surgical Sciences (DIMEC), IRCCS Azienda Ospedaliero – Universitaria di Bologna, Bologna, Italy; 14 Endocrinology Unit, Fondazione IRCCS Ca’ Granda Ospedale Maggiore Policlinico, Milan, Italy; 15 Pediatric Section, Department of Translational Medical Sciences, University of Naples Federico II, Naples, Italy; 16 Health Sciences Department, University of Florence, Florence, Italy; 17 Meyer Children’s Hospital IRCCS, Florence, Italy; 18 Pediatric Unit, IRCCS Azienda Ospedaliero – Universitaria di Bologna, Bologna, Italy; 19 Section of Endocrinology, Geriatrics and Internal Medicine, Department of Medical Sciences, University of Ferrara, Ferrara, Italy

**Keywords:** Delphi consensus, Multidisciplinary care, Patient-centered approach, Transition management, Turner syndrome

## Abstract

**Purpose:**

Transition from pediatric to adult care is associated with significant challenges in patients with Turner syndrome (TS). The objective of the TRansition Age Management In Turner syndrome in Italy (TRAMITI) project was to improve the care provided to patients with TS by harnessing the knowledge and expertise of various Italian centers through a Delphi-like consensus process.

**Methods:**

A panel of 15 physicians and 1 psychologist discussed 4 key domains: transition and referral, sexual and bone health and oncological risks, social and psychological aspects and systemic and metabolic disorders.

**Results:**

A total of 41 consensus statements were drafted. The transition from pediatric to adult care is a critical period for patients with TS, necessitating tailored approaches and early disclosure of the diagnosis to promote self-reliance and healthcare autonomy. Fertility preservation and bone health strategies are recommended to mitigate long-term complications, and psychiatric evaluations are recommended to address the increased prevalence of anxiety and depression. The consensus also addresses the heightened risk of metabolic, cardiovascular and autoimmune disorders in patients with TS; regular screenings and interventions are advised to manage these conditions effectively. In addition, cardiac abnormalities, including aortic dissections, require regular monitoring and early surgical intervention if certain criteria are met.

**Conclusions:**

The TRAMITI consensus statement provides valuable insights and evidence-based recommendations to guide healthcare practitioners in delivering comprehensive and patient-centered care for patients with TS. By addressing the complex medical and psychosocial aspects of the condition, this consensus aims to enhance TS management and improve the overall well-being and long-term outcomes of these individuals.

**Supplementary Information:**

The online version contains supplementary material available at 10.1007/s40618-024-02315-4.

## Plain language summary

The TRansition Age Management in Turner syndrome in Italy (TRAMITI) project aims to improve care for individuals with Turner Syndrome (TS) during their transition from pediatric to adult care. A team of 15 physicians and 1 psychologist collaborated to create a comprehensive set of 41 consensus statements, covering four key areas: transition and referral, sexual and bone health and oncological risks, social and psychological aspects and systemic and metabolic disorders. The consensus statements highlight the importance of patient-centered care, early intervention and long-term monitoring. They emphasize a multidisciplinary approach to address the complex medical and psychosocial aspects of TS. During the critical transition period, tailored approaches and early disclosure of the diagnosis are recommended to promote self-reliance and healthcare autonomy. To mitigate long-term complications, the consensus addresses fertility preservation and bone health strategies. It also recommends psychological or psychiatric evaluations to tackle the increased prevalence of anxiety and depression in patients with TS. In addition, strategies for addressing the heightened risk of metabolic, cardiovascular and autoimmune disorders in patients with TS are proposed. Regular screenings and interventions are advised to effectively manage these conditions. Furthermore, cardiac abnormalities, including aortic dissections, require close monitoring and early surgical intervention if specific criteria are met. Overall, the TRAMITI consensus statement provides valuable insights and evidence-based recommendations. It offers guidance for healthcare practitioners in delivering comprehensive and patient-centered care for individuals with TS. By addressing both medical and psychosocial aspects, the consensus aims to enhance TS management and improve the well-being and long-term outcomes of those affected by this genetic disorder.

**Supplementary Information:**

The online version contains supplementary material available at 10.1007/s40618-024-02315-4.

## Introduction

Turner syndrome (TS) is a genetic disorder characterized by the complete or partial loss of one of the two X chromosomes, affecting only women at an approximate rate of 1 in 2500 live births [1, 2]. The characteristic clinical features of TS include short stature, webbed neck, cardiac defects, endocrine and metabolic disorders and infertility [3, 4].

TS management poses unique challenges due to multisystem involvement, necessitating a coordinated multidisciplinary approach to ensure optimal patient care [5]. Regular monitoring and timely interventions across various medical specialties including cardiology, endocrinology and psychology are essential to mitigate the long-term complications of TS and improve quality of life (QoL) [5].

One of the most significant challenges in TS management is the transition from pediatric to adult care [6, 7]. This transition is a critical period as individuals with TS experience numerous physiological and psychosocial changes that require a shift in healthcare priorities [6]. Although a number of transition protocols have been proposed, many patients with TS do not receive appropriate care during this critical period, predisposing them to adverse health outcomes and threatening their QoL [8, 9].

Given the multifaceted challenges associated with TS, there is a need for comprehensive consensus about the transition of patients from pediatric to adult healthcare. The objective of the TRansition Age Management In Turner syndrome in Italy (TRAMITI) project was to improve the care provided to patients with TS at the national level by harnessing the experiences of various Italian centers through a Delphi-like consensus process.

## Methods

The TRAMITI project employed a Delphi-like methodology. Members of the TRAMITI working group included 15 Italian physicians and 1 psychologist who are all experts in TS management, including both pediatric and adult endocrinologists, a cardiologist and a gynecologist. Members of the working group were selected based on their level of experience in their respective specializations (i.e., pediatric or adult), and in transitioning patients with TS from pediatric to adult care.

The initial advisory board meeting for the project was convened in Rome on 21 December 2022. The primary objectives of this meeting were to establish the rationale and structure of the project, identify key topics and subtopics for consensus statements, delineate the roles and responsibilities of working subgroups and outline a methodology for these subgroups.

Prior to the meeting, a comprehensive literature search was conducted using Boolean operators and keywords relevant to various topics under consideration (Supplemental Methods; Online Resource 1). The results of this search were disseminated among the participants, ensuring that a foundational set of literature was readily available for reference throughout the project. Members of the advisory board could also suggest references.

Following extensive discussion, members of the advisory board agreed that the consensus statements should encompass four topics: transition and referral, sexual and bone development, systemic and metabolic disorders and social and psychological aspects (Supplemental Methods; Online Resource 1). Members also agreed to constitute four working subgroups in such a way as to ensure an equitable distribution of members across various specialties (Supplemental Methods; Online Resource 1). Each working subgroup was tasked with independently reviewing literature pertinent to their specific topic, developing shared insights and generating statements and arguments based on these insights. Working subgroups were advised to use the modified Grading of Recommendations, Assessment, Development, and Evaluation (GRADE) system [10, 11]. In the GRADE system, 1 indicates a strong recommendation and 2 indicates a weak recommendation. Quality of evidence is classified as +00 (low), ++0 (moderate) or +++ (high).

Following the initial meeting, each working subgroup held two asynchronous meetings. The first meeting, entitled ‘Problem setting and analysis of the subtopic,’ involved the review of the relevant literature, development of the subtopics and drafting of statements and supporting arguments. The first set of asynchronous meetings took place in February 2023. The second asynchronous meeting, entitled ‘Definition of the statements for the subtopic,’ encompassed discussion of the statements to clarify their wording, as well as any remaining points, and the finalization of the statements to be shared with the entire advisory board. The second set of asynchronous meetings took place in March 2023. Working subgroups were given the following criteria for drafting the statements: the statements should be in English, be affirmative, not contain double negatives, be concise, focused on a single theme and be supported by references.

The final meeting was held in Bologna on 27 April 2023. During this meeting, the work of each subgroup was consolidated, the statements and the arguments were reviewed collectively and, if necessary, refined. After that, members of the advisory board voted on each statement online selecting the options ‘agree’ or ‘do not agree.’

## Results

A total of 41 statements were drafted and subject to a vote (Table 1). All 16 members of the working group agreed with 39 statements. Fifteen members of the working group agreed with statement B1, while one member abstained. Similarly, 15 members of the working group agreed with statement D9 and one member disagreed.

**Table 1:** Final consensus statements

No	Statement	Rating
*Working subgroup A: transition and referral*		
A1	If possible, TS patients should be transitioned from pediatric to adult endocrinologist at the age of 18 years	1/++0
A2	The appropriate age could differ depending on the readiness of each patient	1/++0
A3	A validated assessment questionnaire should be used to evaluate transition readiness in TS patients	1/++0
A4	To ensure that patients with TS are physically fit for transition, milestones for the health management in adulthood should have been achieved	1/++0
A5	Adolescent patients with TS should be informed about all aspects of the syndrome during adult life, including the risk of complications and the need for regular follow-up and preventive healthcare	1/++0
A6	Before transition, patients' and caregivers' knowledge about TS should be reviewed, including the need and willingness for any additional education, which should be personalized	1/++0
A7	During the transition process, telemedicine can enhance communication and the sharing of information and increase flexibility in visit scheduling	2/+00
A8	Ideally, transition to adult care for TS patients should take place at the same hospital where the patient was treated by a pediatrician in the setting of a multidisciplinary team	1/++0
A9	A medical record summary containing essential TS-specific clinical information is necessary during transition from the care of pediatric to adult endocrinologist	1/++0
A10	The first transition visit should be managed by both pediatric and adult endocrinologists as an opportunity to ensure a continuity of care and to establish a comprehensive care plan	1/++0
*Working subgroup B: sexual and bone health and oncological risk*		
B1	Most girls with TS require HRT, initially for the induction of puberty and later for maintaining secondary sex characteristics, attaining peak bone mass and normalizing uterine growth for possible pregnancy	1/++0
B2	Transdermal preparations of ERT are the preferred regimen in girls with TS	1/+00
B3	Girls with TS typically have a normal uterus, and progestin/progesterone must be added once breakthrough bleeding occurs or after 2 years of ERT	1/+00
B4	During transition, or before the transition process begins, individuals with TS should be counseled that their ability to conceive spontaneously decreases rapidly with age and may not be present during adulthood and that spontaneous pregnancies are rare	1/+++
B5	Fertility assessment should be performed between the age of 12 and 14 years; the best procedure for preserving fertility in young women with TS and persistent ovarian function is oocyte or embryo cryopreservation	2/+00
B6	Osteopenia and osteoporosis are common features in young women with TS, with an estimated prevalence of 50% and an increased risk of early bone fractures	1/++0
B7	ERT is essential for the maintenance of bone health in TS, and early initiation is one of the most important determinants of bone health	1/+++
B8	Transdermal estrogens appear to have more beneficial effects on bone mass than oral preparations	2/+00
B9	Although overall oncologic risk in TS is similar or slightly higher compared with the general population, particular attention should be paid to the monitoring for specific tumors, such as thyroid carcinoma, meningioma and skin tumors	1/+00
B10	Individuals with a Y chromosome may be at risk of gonadoblastoma, therefore gonadectomy should be discussed with these patients and their caregivers, taking into account the balance of the risks and benefits of the procedure	2/+00
*Working subgroup C: social and psychological aspects*		
C1	For an effective transition process, direct involvement of the patient with TS in the treatment path is essential	1/++0
C2	TS is associated with the presence of a characteristic neurocognitive profile that can influence the long-term adaptive and social functioning and the acquisition of full autonomy	1/++0
C3	TS is associated with psychiatric disorders, including a significantly elevated risk of anxiety and depression in adolescents and young adults	1/++0
C4	Anxiety and depression interfere with the psychological health and function of patients with TS	1/++0
C5	A periodic neuropsychological assessment during the age of development is needed to prevent the occurrence or worsening of psychopathological problems later in life	1/++0
C6	Psychodiagnostic assessment should be reinforced in adolescence and at the age of transition in patients with TS	1/++0
C7	Sexuality-related attitudes and behaviors of adolescents with TS should be discussed before the transition to adult care to ensure greater psycho-relational well-being	1/++0
C8	Adolescents and young women with TS have a lower QoL than the general population, with a marked impairment in psychosocial variables, although generally their socioeconomic status does not seem to be impaired	1/++0
C9	Since eating disorders are more frequent in adolescents with TS compared with the general population, this issue should be proactively investigated	1/++0
*Working subgroup D: systemic and metabolic disorders*		
D1	In young women, screening for DM using HbA1c and FPG is recommended annually, and with OGTT every two years, regardless of BMI, family history, karyotype, previous therapy with recombinant GH and therapy with estrogens/progestins	1/++0
D2	BMI is not sufficiently reliable to identify TS subjects with obesity, therefore waist circumference, waist-to-height ratio and body composition assessment are recommended annually	1/++0
D3	Annual screening for liver function tests is recommended, and liver ultrasound should be performed if they are markedly and persistently altered. HRT should not be interrupted because of liver dysfunction	1/++0
D4	At least one ambulatory BP assessment per year is required and, at the first diagnosis of hypertension, secondary causes must be excluded. It is reasonable that Holter BP measurement is performed at least once	1/+++
D5	As antihypertensive drugs β-blockers, ARBs or ACE-I should be used in first-line treatment. In addition, losartan may be effective in reducing aortic growth velocity	2/+00
D6	During transition, TTE or cardiac magnetic resonance CMR assessment studies should be performed and tailored to each patient in case of structural heart disease	1/++0
D7	Patients' awareness of the risk of aortic dissections and the early recognition of symptoms are essential	1/++0
D8	Surgical management of the aortic root and ascending aorta is recommended for women with TS aged ≥ 15 years, ascending ASI ≥ 25 mm/m^2^, regardless of the presence of risk factors for aortic dissection	2/++0
D9	The assessment of anti-TPO antibodies is recommended annually, until positive results. The assessment of TSH ± FT4 is recommended annually, regardless of the presence of Hashimoto’s thyroiditis or L-thyroxine replacement therapy	1/+++
D10	At transition, thyroid ultrasound should be performed and repeated every two years in cases of altered thyroid function or positive Anti-TPO Ab, or annually in case of thyroid nodules and/or goiters	2/++0
D11	Screening for celiac disease should be performed once every 3 years or earlier in case of clinical suspicion. Autoimmunity screening for the other diseases should be performed only in case of clinical suspicion	1/++0
D12	Audiologic and sensorineural screening should be periodically performed in all patients to monitor hearing loss and other complications, and to guarantee psychosocial integration and improved QoL	1/++0

*ACE-I* angiotensin-converting enzyme inhibitor; *ARB* angiotensin receptor blocker; *ASI* aortic size index; *BMI* body mass index; *CMR* cardiac magnetic resonance; *DM* diabetes mellitus; *ERT* estrogen replacement therapy; *FT4* free thyroxine; *FPG* fasting plasma glucose; *GH* growth hormone; *HbA1c* glycated hemoglobin; *HRT* hormone replacement therapy; *OGTT* oral glucose tolerance test; *QoL* quality of life; *TPO* thyroid peroxidase; *TS* Turner syndrome; *TSH* thyroid-stimulating hormone; *TTE* trans-thoracic echocardiography

### Transition and referral

The transition process and its timing should be tailored to the unique needs of young adults with TS and should be based on interdisciplinary collaboration between pediatric and adult care specialists [5]. Ideally, transition should occur around the age of 18, as timely integration into an adult clinic is predictive of consistent and stable long-term follow-up (Statement A1) [12].

Early disclosure of TS diagnosis to parents and, subsequently, to the patient around the age of 11–13 years, promotes self-reliance and healthcare autonomy [5, 13]. Developmental age should take precedence over chronological age in determining communication timing (Statement A2) [14].

Since there is currently no validated TS-specific transition readiness assessment tool, the Transition Readiness Assessment Questionnaire (TRAQ) 5.0 could be recommended due to its well-defined structure and ease of use [15] (Statement A3). TRAQ is a validated instrument comprising 20 items designed to evaluate an individual's knowledge and self-assessed competencies in health-related skills [16].

In order to reduce the risk of chronic complications and comorbidities in women with TS, a timely initiation of hormone replacement therapy (HRT) for ovarian dysfunction, continued management of complications that began in childhood and proactive screening for comorbidities that may emerge in adulthood are necessary (Statement A4) [5, 17–19]. Adolescents with TS should be informed about how their condition is likely to affect their adult lives, about the risk of complications and the need for regular follow-up and preventive care (Statement A5) [5].

Identifying disease-specific knowledge is crucial due to the significance of self-efficacy in behavioral interventions [5, 20]. Before transitioning, a comprehensive review of TS-related issues and future care plans is essential (Statement A6). Transition discussions should encompass the adolescent’s progression into adulthood, including health, independence, education, career and social aspects [5, 7, 12, 17, 21, 22]. Psychological support, educational assistance and engagement with TS support groups are all invaluable in boosting self-esteem and essential skills [5, 7, 12, 17, 21, 22].

Telemedicine can be helpful in enhancing the sharing of information between patients and clinical staff, thus bolstering the effectiveness of consultations and adherence to follow-up appointments (Statement A7). Incorporating telemedicine platforms into transition strategies offers the potential for greater flexibility in scheduling visits and mitigating social and logistical hurdles for both patients and parents [13, 23, 24]. However, it is important to recognize that telemedicine should complement, rather than supplant, in-person visits.

Various models of transition have been proposed, ranging from vertically integrated care within a single hospital to care provided across different sites within the same healthcare system [7, 25]. Ideally, transition should take place in the same hospital where the patient received pediatric care (Statement A8). An essential element of transition is a medical record summary that includes TS-specific clinical information, which should be shared by the pediatric and adult endocrinologists with the patient and their caregivers (Statement A9) [26]. The Endocrine Society offers a specialized tool, the Clinical Summary and Transfer Record for Youth with Turner Syndrome, which can be utilized as a TS-specific transition aid [15].

Both pediatric and adult endocrinologists are regarded as the driving forces within the multidisciplinary care team [13]; the first transition visit should be managed by both specialists (Statement A10).

### Sexual health and fertility

TS is usually accompanied by hypergonadotropic hypogonadism and amenorrhea [5, 27, 28]. As a result, spontaneous puberty occurs in 21–50% of TS girls and menarche in 15–30% of patients with mosaicism [5, 27, 28]. Thus, pubertal failure is frequent in girls with TS, and most need hormonal replacement therapy (HRT) for induction or completion of puberty, maintenance of secondary sex characteristics, attaining peak bone mass and normalizing uterine growth for potential pregnancies (Statement B1). To mimic physiological initiation and/or progression of puberty, treatment should begin at 11–12 years of age, or whenever pubertal development fails to progress [27].

Although the optimal regimen for estrogen replacement therapy (ERT) in girls with TS has not been established [27], transdermal preparations of estradiol should be the preferred choice (Statement B2). In fact, ERT administered via a systemic route tends to mimic physiological estradiol secretion more closely. In contrast, when given orally estrogen reaches the systemic circulation only after absorption through the portal venous system, thus exposing the liver to a greater amount of estrogen than the rest of the body [27]. However, the oral route might be the preferred choice from the patients’ perspective and often girls ask for an oral estro-progestin pills as maintenance HRT at the end of induction.

Girls with TS usually have a normal uterus, mandating the addition of progestin after 2 years of ERT or on the occurrence of breakthrough bleeding (Statement B3) [5]. Although a 10-day treatment cycle and natural progesterone preparations are preferred, there is a paucity of evidence to support these options [5, 28].

Fertility is an area of considerable concern for individuals with TS [5], and thus fertility counseling should be undertaken as soon as diagnosis is confirmed. At transition, fertility counseling should be repeated, or conducted if it has not been undertaken before. Those with TS should be counseled that their ability to conceive decreases with age and that spontaneous pregnancies are rare (Statement B4) [5]. A fertility assessment should be performed at transition, even if conducted previously, and periodically thereafter until premature ovarian insufficiency occurs (Statement B5). However, in prepubertal girls with TS a reliable marker predicting absent pubertal ovarian activity and thus indicating the need for precocious fertility preservation is not available, even if some evidence may suggest that undetectable anti-Mullerian hormone levels predict absent pubertal onset [29]. Young women with TS and persistent ovarian function should be counseled that oocyte cryopreservation after controlled ovarian hyperstimulation or embryo cryopreservation are the best options available to preserve fertility (Statement B5) [30, 31]. Ovarian tissue cryopreservation (OTC) is an established option for those undergoing treatments that can damage the ovaries (Statement B5) and may represent the only option for prepubertal TS patients, although it should still be regarded as experimental [32]. To date, two pregnancies have been reported in patients with TS after implantation of ovarian tissue, one following autografting of ovarian tissue donated by her monozygotic twin and, more recently, a clinical pregnancy conceived naturally after re-implantation of cryopreserved ovarian tissue removed soon after spontaneous puberty [33, 34]. In addition to surgical risks and ethical issues, removal of one or part of an ovary may cause earlier premature ovarian insufficiency. All these issues, in the light of current knowledge, should be discussed with the prepubertal adolescent and her family. Since fertility preservation procedures are also invasive, potentially fraught with complications and costly, patients with TS should also be counseled about alternative paths to parenthood.

### Bone health

Osteopenia and osteoporosis affect approximately 50% of women with TS (Statement B6) [35, 36]. Patients with TS have a 25% increased fracture risk compared with the general population, predominantly seen during childhood and after 45 years [37, 38]. Dual-energy X-ray absorptiometry (DXA) is the gold standard for assessment of bone mineral density (BMD), although it has some limitations in TS patients. Areal BMD values obtained by DXA are influenced by bone size and short stature, thus resulting in an underestimation of BMD. Healthy lifestyle advice, including on physical activity and nutrition, should be given to patients with TS, and patients with TS should be screened for vitamin D deficiency between 9 and 11 years of age, and every 2–3 years thereafter [39].

ERT plays a pivotal role in preserving bone health in individuals with TS (Statement B7) [40–44]. The timing of ERT initiation is crucial in safeguarding bone health in patients with TS [40, 42–44]. It appears that the use of transdermal estrogens may have a more favorable impact on bone mass compared with oral administration (Statement B8) [45].

### Oncological risk

There is a lack of studies specifically focusing on cancer risk during the transition age in TS, with existing information primarily derived from large-scale population-based studies, which encompass patients within the transition age range [46–50]. Standardized incidence ratios range from 0.9 to 1.82, with increased risk of benign central nervous system tumors and skin neoplasms, and a reduced risk for breast cancer [46–50]. HRT and growth hormone treatments do not seem to affect overall cancer risk, including breast cancer, in females with TS, however, it is important to closely monitor for specific types of tumors such as thyroid carcinoma, meningioma and skin tumors (Statement B9) [49].

International guidelines advocate for prophylactic gonadectomy in patients with TS with a Y chromosome (TS + Y) upon diagnosis [5, 51]. However, only one national registry reported an increased risk of gonadoblastoma by the age of 25 in such individuals [47], and the quality of evidence behind this recommendation is rather low. Most of the germ cell tumors in patients with TS + Y described thus far are based on a small number of case reports and case series with likely publication bias, and the optimal strategy for screening and performing gonadectomy in these patients remains unclear. Cases of gonadoblastoma have been described in children (two reports of dysgerminoma at 6 and 10 years old) but generally malignant transformation occurs in older patients in their second decade [51]. A recent multicenter study evaluating 44 patients with TS + Y (19 patients aged ≥ 13 years) found that 42% of girls entered puberty spontaneously and 11% had spontaneous menarche, supporting gonadal function [52]. Gonadoblastoma was identified in seven patients (19%), one patient had in situ germ cell neoplasia and one patient had dysgerminoma (3%) [52]. In addition, a number of reports of spontaneous pregnancies and live births have been published, demonstrating that some women with TS + Y have gonadal function [53–55]. As fertility preservation methods improve, the potential for fertility in patients with TS + Y needs to be weighed against the risk of gonadal malignancy, and discussions regarding gonadectomy conducted with these patients and their caregivers (Statement B10) [53, 54].

### Social and psychological aspects

Although historically physicians have often withheld information from patients with sex development disorders (including TS), the current guidelines advocate for patients’ rights to be informed of their diagnosis [5, 56, 57]. Open and transparent communication with physicians is an essential part of transitioning from pediatric to adult care for patients with TS as it promotes self-awareness and identity formation [56]. The Chicago Consensus Statement underscores the importance of gradual disclosure tailored to the patient's cognitive and emotional capacities [57]. Involvement of the patient in their own treatment path is fundamental (Statement C1).

Individuals with TS typically have normal IQ levels but exhibit deficits in visual-spatial reasoning, information processing speed, working memory, executive functions and social cognition, which impact their daily life [58–60]. These deficits also significantly affect academic performance, social interactions and occupational functioning (Statement C2) [58–60]. Although HRT can improve some aspects of cognitive functioning, social competencies remain affected [58].

There is an increased prevalence of psychiatric disorders, especially anxiety and depression, in individuals with TS (Statement C3) [61–64]. Cultural factors, physiological aspects and social interactions influence emotional well-being in adults with TS [65]. Anxiety and depression impair the psychological health and functioning of patients with TS (Statement C4), with anxiety exacerbating attention challenges [3].

Routine neuropsychological assessments and psycho-diagnostic evaluations aimed at detecting and treating critical neurocognitive issues are essential in early identification of psychological and emotional problems (Statement C5), especially in adolescents transitioning to adulthood (Statement C6) [66]. Standardized questionnaires and psychometric tools are recommended for screening symptoms of anxiety and depression, and any detected symptoms should prompt further assessment by mental health specialists [63, 67].

Addressing sexuality-related attitudes and behaviors in adolescents with TS prior to transitioning to adult care is vital for improved psychosocial well-being (Statement C7). Inducing puberty in alignment with physiological norms is crucial, and psychological support is especially important for patients from low socioeconomic backgrounds [68, 69].

Patients with TS often have lower QoL, particularly in psychosocial aspects, despite maintaining a stable socioeconomic status (Statement C8) [70, 71]. Life satisfaction is influenced by personal factors, and attention to self-perception and health-related sentiments is critical for improving QoL [72].

There is an increased prevalence of eating disorders among adolescents with TS, and aspects such as negative self-image, immature behavior, delayed pubertal development and HRT may contribute to their etiology (Statement C9) [73, 74].

### Systemic and metabolic disorders

Patients with TS frequently have various metabolic, cardiovascular and autoimmune disorders.

TS is associated with an increased incidence and prevalence of diabetes mellitus (DM) [75–78]. DM in patients with TS is primarily caused by impaired insulin secretion due to autoimmunity or other reasons that are currently unknown [76, 77]. The time-course of insulin, glucose, and insulinogenic index in response to glycemic challenge do not differ between antibody-positive and antibody-negative individuals with DM [79]; therefore, screening for autoimmune diabetes is not recommended until DM is diagnosed. The available evidence indicates that the oral glucose tolerance test (OGTT) is superior to fasting glucose and glycated hemoglobin (HbA1c) in early DM diagnosis [77, 78, 80]. Therefore, an annual HbA1c and fasting glucose screening and a biennial OGTT screening are recommended (Statement D1).

Obesity is common in patients with TS, who have a distinct body fat distribution, characterized by increased visceral fat and reduced subcutaneous fat and lean body mass compared with women without TS [81–86]. Therefore, body mass index (BMI) is not an effective measure of adiposity in this population and alternative measures, such as waist circumference, waist-to-height ratio and body composition analysis, should be used (Statement D2) [86].

Hepatic abnormalities, including architectural changes, alterations in liver enzymes and hepatic steatosis, are common in TS [87–91]. Their cause remains uncertain, but congenital vascular disorders, metabolic syndrome and estrogen deficiency, environmental influences, lifestyle, genetic and epigenetic factors, may play a role [89, 91]. In addition, biliary conditions, including sclerosing cholangitis, primary biliary cholangitis, biliary atresia and a paucity of bile ducts, can develop, primarily as a result of primary hepatic vascular abnormalities [89, 92]. Liver function should be assessed annually in patients with TS and an ultrasound should be performed in case of significant and consistent abnormalities (Statement D3). Discontinuation of HRT is unwarranted as it does not cause deterioration of liver function [89, 91].

Patients with TS often have hypertension, affecting up to 25% of adolescents and between 13 and 58% of adults, and influenced greatly by factors such as race and lifestyle [93–97]. Hypertension in TS is predominantly characterized by an elevated systolic pressure and the absence of the typical circadian variation, with abnormal nocturnal dips observed as early as infancy [93, 97]. Patients with TS should undergo at least one ambulatory blood pressure assessment annually (Statement D4). Upon an initial diagnosis of hypertension, secondary causes should be meticulously investigated and ruled out. Beta-blockers, angiotensin receptor blockers (ARBs) or angiotensin-converting enzyme inhibitors (ACE-I) are recommended as first-line treatment for hypertension in patients with TS, while losartan can be used to reduce the growth rate of the aorta (Statement D5).

Approximately, 30% of patients with TS have congenital cardiac anomalies, with the most common being a bicuspid aortic valve and coarctation of the aorta [94, 98–100]. These anomalies are observed more frequently in those with 45X monosomy compared with those with mosaicism and often contribute to early mortality even after surgical or percutaneous intervention [98, 99, 101, 102]. In patients with TS, electrocardiogram (ECG) abnormalities such as QT interval prolongation have been reported starting from childhood, although they are generally mild in severity [94, 103].

Aortic dilation in TS is serious and often leads to fatal dissections [94, 104–106]. Typically originating in the ascending aorta, aortic dissections account for 2–8% of deaths in patients with TS [94, 107]. The condition occurs at an average age of 35 in those with TS, as opposed to 68 in the general population [94, 104, 108]. Aortic size index (ASI) is the primary parameter for assessing the risk of dissection [109]. During the transition phase, it is imperative for patients with TS to undergo trans-thoracic echocardiography (TTE) or cardiac magnetic resonance (CMR) assessments, as these methods provide high accuracy in measuring aortic size (Statement D6) [100, 110]. Educating affected patients about the risks of aortic dissections and the importance of early symptom recognition is essential (Statement D7). For those with TS aged ≥ 15 years, it is advisable to undertake surgical intervention for the aortic root and ascending aorta if ascending ASI is ≥ 25 mm/m^2^, irrespective of the presence of risk factors for aortic dissection (Statement D8).

Autoimmune disorders are also prevalent in TS due to complex genetic and environmental interactions [111–113]. Hashimoto’s thyroiditis is common, affecting approximately 50% of individuals with TS [111–113]. Moreover, a Danish study revealed that 57% women with TS had at least one type of autoantibody, most commonly anti-thyroid peroxidase antibodies (TPO), which is significantly higher than in the general population [111]. The annual evaluation of anti-TPO antibodies is advisable for all patients with TS until positive results are obtained. Thyroid-stimulating hormone (TSH) and possibly free thyroxine (FT4) should be annually measured regardless of the presence of Hashimoto’s thyroiditis (Statement D9). In the presence of thyroid nodules and/or goiter, an ultrasound should be conducted annually (Statement D10). Screening for celiac disease should be performed every 3 years, while screening for other autoimmune diseases should be conducted in the presence of clinical indications (Statement D11).

Furthermore, it is imperative to conduct regular audiologic and sensorineural assessments for all patients to keep track of hearing loss and related complications. These assessments are crucial for ensuring psychosocial integration and enhancing QoL (Statement D12).

Reassuringly, HRT does not appear to adversely affect the risk of DM, liver enzyme abnormalities or autoimmune conditions [114–116].

## Conclusions

The TRAMITI project produced a comprehensive set of consensus statements crafted through collaborative efforts of expert Italian physicians specializing in fields related to the management of TS. These statements address four key domains: transition and referral; sexual, bone health and oncological risks; social and psychological aspects; and systemic and metabolic disorders. These consensus statements have the potential to enhance TS management by providing healthcare practitioners with a structured guide that emphasizes patient-centered care, early intervention and long-term monitoring. In addition, by focusing on the needs and psychological well-being of patients with TS, these recommendations address not only the medical, but also the psychosocial aspects that are crucial for improving the QoL and long-term outcomes of these individuals.

### Supplementary Information

Below is the link to the electronic supplementary material.

Supplementary file1 (DOCX 25 KB)

## Data Availability

Data sharing is not applicable to this article as no datasets were generated or analyzed during the current study.
