# Supplementary material for: Transition from pediatric to adult care in patients with Turner syndrome in Italy: a consensus statement by the TRAMITI project
Source: J Endocrinol Invest. 2024 Feb 20;47(7):1585–98. doi: 10.1007/s40618-024-02315-4 (PMC11196323; doi:10.1007/s40618-024-02315-4)
Supplement: Supplementary file 1 — Supplementary file1 (DOCX 25 KB) [file 40618_2024_2315_MOESM1_ESM.docx]

**Transition from pediatric to adult care in patients with Turner syndrome in Italy: a consensus statement by the TRAMITI project**

***Journal of Endocrinological Investigation***

**Electronic Supplementary Material**

**Authors:** Tommaso Aversa,^1,2^ Luisa De Sanctis,^3,4^ Maria Felicia Faienza,^5^ Alessandra Gambineri,^6^ Anna Balducci,^7^ Roberta D’Aprile,^8,9^ Carolina Di Somma,^10^ Claudia Giavoli,^11^ Armando Grossi,^12^ Maria Cristina Meriggiola,^13^ Eriselda Profka,^14^ Mariacarolina Salerno,^15^ Stefano Stagi,^16,17^ Emanuela Scarano,^18^ Maria Chiara Zatelli^19^ and Malgorzata Wasniewska^20,21^

**Affiliations:** ^1^Department of Human Pathology of Adulthood and Childhood, University of Messina, Messina, Italy; ^2^Pediatric Unit, University Hospital “G. Martino,” Messina, Italy;

^3^Pediatric Endocrinology, Regina Margherita Children Hospital, Turin, Italy; ^4^Department of Public Health and Pediatric Sciences, University of Turin, Turin, Italy; ^5^Department of Precision and Regenerative Medicine and Ionian Area, University of Bari “Aldo Moro,” 70121 Bari, Italy; ^6^Division of Endocrinology and Diabetes Prevention and Care, Department of Medical and Surgical Sciences (DIMEC), IRCCS Azienda Ospedaliero – Universitaria di Bologna, Bologna, Italy; ^7^Pediatric Cardiology and Adult Congenital Heart Disease Program, Department of Cardio - Thoracic and Vascular Medicine, IRCCS Azienda Ospedaliero - Universitaria di Bologna, Bologna Italia; ^8^Department of Women's and Children's Health, University of Padua, Padua, Italy; ^9^A.Fa.D.O.C. Association OdV, Vicenza, Italy; ^10^Unit of Endocrinology, AOU Federico II, Naples, Italy; ^11^Endocrinology Unit, Fondazione IRCCS Cà Granda Ospedale Maggiore Policlinico, Milan, Italy; ^12^Endocrine Pathology of Chronic and Post-Tumor Diseases Unit, "Bambino Gesù" Pediatric Hospital, Rome, Italy; ^13^Division of Gynecology and Physiopathology of Reproduction, Department of Medical and Surgical Sciences (DIMEC), IRCCS Azienda Ospedaliero – Universitaria di Bologna, Bologna, Italy; ^14^Endocrinology Unit, Fondazione IRCCS Ca' Granda Ospedale Maggiore Policlinico, Milan, Italy; ^15^Pediatric Section, Department of Translational Medical Sciences, University of Naples Federico II, Naples, Italy; ^16^Health Sciences Department, University of Florence, Florence, Italy; ^17^Meyer Children’s Hospital IRCCS, Florence, Italy; ^18^Pediatric Unit, IRCCS Azienda Ospedaliero – Universitaria di Bologna, Bologna, Italy; ^19^Section of Endocrinology, Geriatrics and Internal Medicine, Department of Medical Sciences, University of Ferrara, Ferrara, Italy; ^20^Department of Human Pathology of Adulthood and Childhood, University of Messina, Italy; ^21^Pediatric Unit, University Hospital “G. Martino,” Messina, Italy.

**Corresponding author:** Malgorzata Wasniewska

**Email:** malgorzata.wasniewska@unime.it

**Supplemental materials**

**Literature search strategy**

The literature search was conducted up to 20 November 2022.

All terms were included in the string both as regular text and as Emtree terms (if present in the Emtree thesaurus) for Embase searches or as MeSH terms (if present in the MeSH database) for PubMed searches. Searches were limited to title, abstract and keywords fields. Other filters used were timeframe (2012–2022) and publication type (all types, except case reports).

***String 1 (Embase)***

(('turner syndrome'/exp OR '45, x syndrome':ti,ab,kw OR 'bonnevie ullrich turner syndrome':ti,ab,kw OR 'bonnevie ullrich state':ti,ab,kw OR 'bonnevie ullrich status':ti,ab,kw OR 'bonnevie-ullrich syndrome':ti,ab,kw OR 'bonneville ullrich turner syndrome':ti,ab,kw OR 'bournevie ullhrich turner syndrome':ti,ab,kw OR 'shereshevsky turner syndrome':ti,ab,kw OR 'turner albright syndrome':ti,ab,kw OR 'turner ullrich syndrome':ti,ab,kw OR 'turner disease':ti,ab,kw OR 'turner stigma':ti,ab,kw OR 'turner syndrome':ti,ab,kw OR 'turner`s syndrome':ti,ab,kw OR 'turners syndrome':ti,ab,kw OR 'ullrich turner syndrome':ti,ab,kw OR 'ullrich syndrome':ti,ab,kw OR 'x-chromosome monosomy':ti,ab,kw OR 'xo turner syndrome':ti,ab,kw OR 'xo syndrome':ti,ab,kw OR 'karyotype 45, x syndrome':ti,ab,kw OR 'karyotype 45, xo syndrome':ti,ab,kw OR 'syndrome, turner':ti,ab,kw OR 'xo male':ti,ab,kw) AND ('transitional age':ti,ab OR 'transit* age*' OR 'transitional care'/exp OR 'transition care':ti,ab OR 'transitional care':ti,ab OR 'transition to adult care'/exp OR 'hand-over to adult care':ti,ab,kw OR 'handoff to adult care':ti,ab,kw OR 'transfer to adult care':ti,ab,kw OR 'transition to adult care':ti,ab,kw OR transition*:ti,ab OR 'patient referral'/exp OR 'gatekeeping':ti,ab,kw OR 'patient referral':ti,ab,kw OR 'referral':ti,ab,kw OR 'referral and consultation':ti,ab,kw OR 'transition* readiness':ti,ab,kw) AND 'patient education'/exp OR 'education, patient':ti,ab,kw OR 'patient education':ti,ab,kw OR 'patient education as topic':ti,ab,kw OR 'patient medication knowledge':ti,ab,kw OR 'caregiver educat*' OR 'education'/exp OR 'child education':ti,ab,kw OR 'education':ti,ab,kw OR 'education service':ti,ab,kw OR 'education, nonprofessional':ti,ab,kw OR 'educational measurement':ti,ab,kw OR 'training support':ti,ab,kw OR 'social barriers':ti,ab OR 'social boundaries':ti,ab OR 'stigma'/exp OR 'stigma':ti,ab,kw OR 'social stigma'/exp OR 'social stigma':ti,ab,kw OR 'social stigmatisation':ti,ab,kw OR 'social stigmatization':ti,ab,kw OR 'stigmatisation':ti,ab,kw OR 'stigmatization':ti,ab,kw OR 'multidisciplinary team'/exp OR 'multi-disciplinary team':ti,ab,kw OR 'multidisciplinary team':ti,ab,kw OR 'interdisciplinary education'/exp OR 'interdisciplinary communication'/exp OR 'nurse patient relationship'/exp OR 'nurse patient relation':ti,ab,kw OR 'nurse patient relationship':ti,ab,kw OR 'nurse-patient relations':ti,ab,kw OR 'patient nurse relation':ti,ab,kw OR 'patient nurse relationship':ti,ab,kw OR 'doctor patient relationship'/exp OR 'bedside psychology':ti,ab,kw OR 'doctor patient contact':ti,ab,kw OR 'doctor patient relation':ti,ab,kw OR 'doctor patient relationship':ti,ab,kw OR 'hospital patient relationship':ti,ab,kw OR 'hospital-patient relations':ti,ab,kw OR 'patient doctor relation':ti,ab,kw OR 'patient doctor relationship':ti,ab,kw OR 'patient physician relation':ti,ab,kw OR 'patient physician relationship':ti,ab,kw OR 'patient staff relation':ti,ab,kw OR 'patient therapist relation':ti,ab,kw OR 'patient therapist relationship':ti,ab,kw OR 'physician patient relation':ti,ab,kw OR 'physician patient relationship':ti,ab,kw OR 'physician-patient relations':ti,ab,kw OR 'psychology, bedside':ti,ab,kw OR 'relation, doctor patient':ti,ab,kw OR 'therapeutic relation':ti,ab,kw OR 'therapist patient relation':ti,ab,kw OR 'therapist patient relationship':ti,ab,kw OR 'professional-patient relationship'/exp OR 'patient-health care professional relation':ti,ab,kw OR 'patient-health care professional relationship':ti,ab,kw OR 'patient-health professional relation':ti,ab,kw OR 'patient-health professional relationship':ti,ab,kw OR 'patient-healthcare professional relationship':ti,ab,kw OR 'patient-professional relations':ti,ab,kw OR 'patient-professional relationship':ti,ab,kw OR 'professional-patient relation':ti,ab,kw OR 'professional-patient relations':ti,ab,kw OR 'professional-patient relationship':ti,ab,kw OR 'long term care'/exp OR 'chronic treatment':ti,ab,kw OR 'life support care':ti,ab,kw OR 'long term care':ti,ab,kw OR 'long term medical care':ti,ab,kw OR 'long term therapy':ti,ab,kw OR 'long term treatment':ti,ab,kw OR 'long-term care':ti,ab,kw OR 'medical care, long term':ti,ab,kw OR 'treatment, long term':ti,ab,kw OR 'chronic therap*':ti,ab OR 'caregiver'/exp OR 'care giver':ti,ab,kw OR 'caregiver':ti,ab,kw OR 'caregivers':ti,ab,kw OR 'carer':ti,ab,kw OR 'carers':ti,ab,kw OR 'family caregiver':ti,ab,kw OR 'family caregivers':ti,ab,kw OR 'caregiver education':ti,ab OR 'education caregiver':ti,ab NOT 'case report'/de AND [2012-2022]/py

***String 2 (Embase)***

'turner syndrome'/exp OR '45, x syndrome':ti,ab,kw OR 'bonnevie ullrich turner syndrome':ti,ab,kw OR 'bonnevie ullrich state':ti,ab,kw OR 'bonnevie ullrich status':ti,ab,kw OR 'bonnevie-ullrich syndrome':ti,ab,kw OR 'bonneville ullrich turner syndrome':ti,ab,kw OR 'bournevie ullhrich turner syndrome':ti,ab,kw OR 'shereshevsky turner syndrome':ti,ab,kw OR 'turner albright syndrome':ti,ab,kw OR 'turner ullrich syndrome':ti,ab,kw OR 'turner disease':ti,ab,kw OR 'turner stigma':ti,ab,kw OR 'turner syndrome':ti,ab,kw OR 'turner`s syndrome':ti,ab,kw OR 'turners syndrome':ti,ab,kw OR 'ullrich turner syndrome':ti,ab,kw OR 'ullrich syndrome':ti,ab,kw OR 'x-chromosome monosomy':ti,ab,kw OR 'xo turner syndrome':ti,ab,kw OR 'xo syndrome':ti,ab,kw OR 'karyotype 45, x syndrome':ti,ab,kw OR 'karyotype 45, xo syndrome':ti,ab,kw OR 'syndrome, turner':ti,ab,kw OR 'xo male':ti,ab,kw) AND ('transitional age':ti,ab OR 'transit* age*' OR 'transitional care'/exp OR 'transition care':ti,ab OR 'transitional care':ti,ab OR 'transition to adult care'/exp OR 'hand-over to adult care':ti,ab,kw OR 'handoff to adult care':ti,ab,kw OR 'transfer to adult care':ti,ab,kw OR 'transition to adult care':ti,ab,kw OR transition*:ti,ab OR 'patient referral'/exp OR 'gatekeeping':ti,ab,kw OR 'patient referral':ti,ab,kw OR 'referral':ti,ab,kw OR 'referral and consultation':ti,ab,kw OR 'transition* readiness':ti,ab,kw) AND (('fertility'/exp OR 'fecundity':ti,ab,kw OR 'fertility':ti,ab,kw OR 'sperm-ovum interactions':ti,ab,kw OR 'infertility'/exp OR 'fertility disorder':ti,ab,kw OR 'infecundity':ti,ab,kw OR 'infertility':ti,ab,kw OR 'primary infertility':ti,ab,kw OR 'secondary infertility':ti,ab,kw OR 'sexual sterility':ti,ab,kw OR 'sterility, sexual':ti,ab,kw OR 'hormone substitution'/exp OR 'hormone replacement':ti,ab,kw OR 'hormone replacement therapy':ti,ab,kw OR 'hormone substitution':ti,ab,kw OR 'substitution, hormone':ti,ab,kw OR hrt:ti,ab OR 'bone health':ti,ab OR 'bone'/exp OR 'bone':ti,ab,kw OR 'bone and bones':ti,ab,kw OR 'cardiovascular risk'/exp OR 'cardiovascular risk':ti,ab,kw OR 'risk, cardiovascular':ti,ab,kw OR 'cardiovascular risk factor'/exp OR 'cardio-vascular risk factor':ti,ab,kw OR 'cardio-vascular risk factors':ti,ab,kw OR 'cardiovascular risk factor':ti,ab,kw OR 'cardiovascular risk factors':ti,ab,kw OR 'risk factor for cardio-vascular disease':ti,ab,kw OR 'risk factor for cardiovascular disease':ti,ab,kw OR 'risk factors for cardio-vascular disease':ti,ab,kw OR 'hypertension'/exp OR 'htn (hypertension)':ti,ab,kw OR 'acute hypertension':ti,ab,kw OR 'arterial hypertension':ti,ab,kw OR 'blood pressure, high':ti,ab,kw OR 'cardiovascular hypertension':ti,ab,kw OR 'controlled hypertension':ti,ab,kw OR 'endocrine hypertension':ti,ab,kw OR 'high blood pressure':ti,ab,kw OR 'high renin hypertension':ti,ab,kw OR 'hypertension':ti,ab,kw OR 'hypertensive disease':ti,ab,kw OR 'hypertensive effect':ti,ab,kw OR 'hypertensive response':ti,ab,kw OR 'neurogenic hypertension':ti,ab,kw OR 'preexistent hypertension':ti,ab,kw OR 'salt high blood pressure':ti,ab,kw OR 'salt hypertension':ti,ab,kw OR 'secondary hypertension':ti,ab,kw OR 'systemic hypertension':ti,ab,kw OR 'obesity'/exp OR 'adipose tissue hyperplasia':ti,ab,kw OR 'adipositas':ti,ab,kw OR 'adiposity':ti,ab,kw OR 'alimentary obesity':ti,ab,kw OR 'body weight, excess':ti,ab,kw OR 'corpulency':ti,ab,kw OR 'fat overload syndrome':ti,ab,kw OR 'nutritional obesity':ti,ab,kw OR 'obesitas':ti,ab,kw OR 'obesity':ti,ab,kw OR 'overweight':ti,ab,kw OR 'cognitive defect'/exp OR 'cognition disorder':ti,ab,kw OR 'cognition disorders':ti,ab,kw OR 'cognitive defect':ti,ab,kw OR 'cognitive defects':ti,ab,kw OR 'cognitive deficit':ti,ab,kw OR 'cognitive disability':ti,ab,kw OR 'cognitive disorder':ti,ab,kw OR 'cognitive disorders':ti,ab,kw OR 'cognitive dysfunction':ti,ab,kw OR 'cognitive impairment':ti,ab,kw OR 'delirium, dementia, amnestic, cognitive disorders':ti,ab,kw OR 'overinclusion':ti,ab,kw OR 'response interference':ti,ab,kw OR 'liver disease'/exp OR 'hepatic disease':ti,ab,kw OR 'hepatic disorder':ti,ab,kw OR 'hepatopathy':ti,ab,kw OR 'liver cell disease':ti,ab,kw OR 'liver disease':ti,ab,kw OR 'liver diseases':ti,ab,kw OR 'liver disorder':ti,ab,kw OR 'liver illness':ti,ab,kw OR 'lifestyle'/exp OR 'life style':ti,ab,kw OR 'lifestyle':ti,ab,kw OR 'quality of life'/exp OR 'hrql':ti,ab,kw OR 'health related quality of life':ti,ab,kw OR 'life quality':ti,ab,kw OR 'quality of life':ti,ab,kw OR 'endocrine disease'/exp OR 'disease, endocrine':ti,ab,kw OR 'endocrinal disorder':ti,ab,kw OR 'endocrine disease':ti,ab,kw OR 'endocrine diseases':ti,ab,kw OR 'endocrine disorder':ti,ab,kw OR 'endocrine disturbance':ti,ab,kw OR 'endocrine dysfunction':ti,ab,kw OR 'endocrine gland disease':ti,ab,kw OR 'endocrine syndrome':ti,ab,kw OR 'endocrine system diseases':ti,ab,kw OR 'endocrinological disease':ti,ab,kw OR 'endocrinopathy':ti,ab,kw OR 'hormonal disorder':ti,ab,kw OR 'hormone dysfunction':ti,ab,kw OR 'hormone imbalance':ti,ab,kw)) NOT 'case report'/de AND [2012-2022]/py

***String 1 (PubMed)***

(("Turner Syndrome"[MeSH Terms] OR "bonnevie ullrich syndrome"[Title/Abstract] OR "status bonnevie ullrich"[Title/Abstract] OR "monosomy x"[Title/Abstract] OR "xo gonadal dysgenesis"[Title/Abstract] OR "gonadal dysgenesis xo"[Title/Abstract] OR "gonadal dysgenesis 45 x"[Title/Abstract] OR "turners syndrome"[Title/Abstract] OR "turner s syndrome"[Title/Abstract] OR "ullrich turner syndrome"[Title/Abstract] OR "syndrome ullrich turner"[Title/Abstract] OR "45 x syndrome"[Title/Abstract] OR "bonnevie ullrich turner syndrome"[Title/Abstract] OR "bonnevie ullrich status"[Title/Abstract] OR "shereshevsky turner syndrome"[Title/Abstract] OR "turner albright syndrome"[Title/Abstract] OR "turner ullrich syndrome"[Title/Abstract] OR "turner disease*"[Title/Abstract] OR "turner stigmata"[Title/Abstract] OR "Turner Syndrome"[Title/Abstract] OR "turner s syndrome"[Title/Abstract] OR "ullrich syndrome"[Title/Abstract] OR "x chromosome monosomy"[Title/Abstract] OR "xo turner syndrome"[Title/Abstract] OR "xo syndrome"[Title/Abstract] OR "syndrome turner"[Title/Abstract] OR "xo male"[Title/Abstract]) AND ("Transitional Care"[MeSH Terms] OR "Transition to Adult Care"[MeSH Terms] OR ("transitional age"[Title/Abstract] OR "transit age*"[Title/Abstract] OR "transition care"[Title/Abstract] OR "Transitional Care"[Title/Abstract] OR "transfer to adult care"[Title/Abstract] OR "Transition to Adult Care"[Title/Abstract] OR "transition*"[Title/Abstract] OR "patient referral"[Title/Abstract] OR "gatekeeping"[Title/Abstract] OR "patient referral"[Title/Abstract] OR "referral"[Title/Abstract] OR "referral and consultation"[Title/Abstract] OR "transition readiness"[Title/Abstract])) AND ("Education"[MeSH Terms] OR "Education"[MeSH Subheading] OR "Social Stigma"[MeSH Terms] OR "Patient Care Team"[MeSH Terms] OR "Nurse-Patient Relations"[MeSH Terms] OR "Professional-Patient Relations"[MeSH Terms] OR "Caregivers"[MeSH Terms] OR "Long-Term Care"[MeSH Terms] OR ("education patient*"[Title/Abstract] OR "patient education"[Title/Abstract] OR "patient knowledge"[Title/Abstract] OR "caregiver educat*"[Title/Abstract] OR "child education"[Title/Abstract] OR "Education"[Title/Abstract] OR "education nonprofessional"[Title/Abstract] OR "nonprofessional education"[Title/Abstract] OR "educational measurement"[Title/Abstract] OR "training support"[Title/Abstract] OR "social barrier*"[Title/Abstract] OR "social boundaries"[Title/Abstract] OR "stigma"[Title/Abstract] OR "Social Stigma"[Title/Abstract] OR "social stigmatisation"[Title/Abstract] OR "social stigmatization"[Title/Abstract] OR "stigmatisation"[Title/Abstract] OR "stigmatization"[Title/Abstract] OR "multi disciplinary team"[Title/Abstract] OR "multidisciplinary team"[Title/Abstract] OR "interdisciplinary"[Title/Abstract] OR "patient health care professional relation*"[Title/Abstract] OR "patient professional relation*"[Title/Abstract] OR "professional patient relation*"[Title/Abstract] OR "chronic treatment"[Title/Abstract] OR "life support care"[Title/Abstract] OR "Long-Term Care"[Title/Abstract] OR "long term medical care"[Title/Abstract] OR "long term therapy"[Title/Abstract] OR "long term treatment"[Title/Abstract] OR "treatment long term"[Title/Abstract] OR "chronic therap*"[Title/Abstract]))) NOT (casereports[Filter]) AND (2012/1/1:3000/12/12[pdat])

***String 2 (PubMed)***

((("Turner Syndrome"[Mesh] OR "bonnevie ullrich syndrome"[Title/Abstract] OR "status bonnevie ullrich"[Title/Abstract] OR "monosomy x"[Title/Abstract] OR "xo gonadal dysgenesis"[Title/Abstract] OR "gonadal dysgenesis xo"[Title/Abstract] OR "gonadal dysgenesis 45 x"[Title/Abstract] OR "turners syndrome"[Title/Abstract] OR "turner s syndrome"[Title/Abstract] OR "ullrich turner syndrome"[Title/Abstract] OR "syndrome ullrich turner"[Title/Abstract] OR "45 x syndrome"[Title/Abstract] OR "bonnevie ullrich turner syndrome"[Title/Abstract] OR "bonnevie ullrich status"[Title/Abstract] OR "shereshevsky turner syndrome"[Title/Abstract] OR "turner albright syndrome"[Title/Abstract] OR "turner ullrich syndrome"[Title/Abstract] OR "turner disease*"[Title/Abstract] OR "turner stigmata"[Title/Abstract] OR "turner syndrome"[Title/Abstract] OR "turner s syndrome"[Title/Abstract] OR "ullrich syndrome"[Title/Abstract] OR "x chromosome monosomy"[Title/Abstract] OR "xo turner syndrome"[Title/Abstract] OR "xo syndrome"[Title/Abstract] OR "syndrome turner"[Title/Abstract] OR "xo male"[Title/Abstract])) AND ((("Transitional Care"[Mesh]) OR "Transition to Adult Care"[Mesh] OR ("transitional age"[Title/Abstract] OR "transit* age*"[Title/Abstract] OR "transition care"[Title/Abstract] OR "transitional care"[Title/Abstract] OR "transfer to adult care"[Title/Abstract] OR "transition to adult care"[Title/Abstract] OR transition*[Title/Abstract] OR "patient referral"[Title/Abstract] OR "gatekeeping"[Title/Abstract] OR "patient referral"[Title/Abstract] OR "referral"[Title/Abstract] OR "referral and consultation"[Title/Abstract] OR "transition readiness"[Title/Abstract])))) AND (((((((((((((((("Fertility"[Mesh]) OR ("fertility"[Title/Abstract])) OR ("fecundity"[Title/Abstract])) OR ("sperm ovum interaction*"[Title/Abstract])) OR ('sperm ovum interaction[Title/Abstract])) OR ((((("Infertility"[Mesh]) OR ("infertility"[Title/Abstract])) OR ("fertility disorder*"[Title/Abstract])) OR ("infecundity"[Title/Abstract])) OR ("sexual sterility"[Title/Abstract]))) OR (((((("Hormone Replacement Therapy"[Mesh]) OR ("hormone replacement therap*"[Title/Abstract])OR ("hormone substitution*"[Title/Abstract])) ) OR ("hormone replacement"[Title/Abstract])) OR ("substitution hormone"[Title/Abstract] OR "substitution hormonotherapy"[Title/Abstract])) OR ("hrt"[Title/Abstract]))) OR (((("bone health"[Title/Abstract]) OR ("bone*"[Title/Abstract])) OR ("bone and bones"[Title/Abstract])) OR ("Bone and Bones"[Mesh]))) OR ((Heart Disease Risk Factor*[Title/Abstract]) OR (((((((("Cardiovascular Diseases"[Mesh]) OR "Heart Disease Risk Factors"[Mesh]) OR ("cardiovascular risk*"[Title/Abstract])) OR ("risk cardiovascular"[Title/Abstract])) OR ("cardio vascular risk factor*"[Title/Abstract])) OR ("cardiovascular risk factor*"[Title/Abstract])) OR ("risk factor for cardiovascular disease*"[Title/Abstract])) OR ("risk factors for cardiovascular disease*"[Title/Abstract])))) OR ((((((((((((((("Hypertension"[Mesh]) OR ("hypertension"[Title/Abstract])) OR ("htn"[Title/Abstract])) OR ("acute hypertension"[Title/Abstract])) OR ("arterial hypertension"[Title/Abstract])) OR ("blood pressure high"[Title/Abstract])) OR ("blood pressures high"[Title/Abstract])) OR ("high blood pressure*"[Title/Abstract])) OR ("cardiovascular hypertension*"[Title/Abstract])) OR ("endocrine hypertension*"[Title/Abstract])) OR ("hypertensive disease*"[Title/Abstract])) OR ("hypertensive effect*"[Title/Abstract])) OR ("hypertensive response*"[Title/Abstract])) OR ("neurogenic hypertension*"[Title/Abstract])) OR ("systemic hypertension*"[Title/Abstract]))) OR (("Obesity"[Mesh]) OR ("obesity"[Title/Abstract] OR "adipose tissue hyperplasia"[Title/Abstract] OR "adipositas"[Title/Abstract] OR "adiposity"[Title/Abstract] OR "alimentary obesity"[Title/Abstract] OR "body weight, excess"[Title/Abstract] OR "corpulency"[Title/Abstract] OR "fat overload syndrome"[Title/Abstract] OR "nutritional obesity"[Title/Abstract] OR "obesitas"[Title/Abstract] OR "overweight"[Title/Abstract]))) OR ((("Cognitive Dysfunction"[Mesh]) OR ("cognition disorder*"[Title/Abstract] OR "cognitive defect*"[Title/Abstract] OR "cognitive deficit"[Title/Abstract] OR "cognitive disability"[Title/Abstract] OR "cognitive disorder*"[Title/Abstract] OR "cognitive dysfunction*"[Title/Abstract] OR "cognitive impairment"[Title/Abstract] OR "overinclusion"[Title/Abstract] OR "response interference"[Title/Abstract])) OR (("cognitive impairment*"[Title/Abstract]) OR ("impairment cognitive"[Title/Abstract]) OR ("impairments cognitive"[Title/Abstract]) OR ("neurocognitive disorder*"[Title/Abstract]) OR ("cognitive decline"[Title/Abstract]) OR ("decline cognitive"[Title/Abstract]) OR ("declines cognitive"[Title/Abstract]) OR ("deterioration mental"[Title/Abstract]) OR ("mental deterioration"[Title/Abstract])))) OR ((("Liver Diseases"[Mesh]) OR "Endocrine System Diseases"[Mesh]) OR ("hepatic disease*"[Title/Abstract] OR "hepatic disorder*"[Title/Abstract] OR "hepatopathy"[Title/Abstract] OR "liver cell disease*"[Title/Abstract] OR "liver disease*"[Title/Abstract] OR "liver disorder*"[Title/Abstract] OR "liver illness"[Title/Abstract] OR "Disease Liver"[Title/Abstract] OR "Diseases Liver"[Title/Abstract] OR "Liver Dysfunction*"[Title/Abstract] OR "Dysfunction Liver"[Title/Abstract] OR "Dysfunctions Liver"[Title/Abstract]))) OR ((("Life Style"[Mesh]) OR ("life style"[Title/Abstract])) OR ("lifestyle*"[Title/Abstract]))) OR ((((("Quality of Life"[Mesh]) OR ("quality of life"[Title/Abstract])) OR ("life quality"[Title/Abstract])) OR ("health related quality of life"[Title/Abstract])) OR ("hrqol"[Title/Abstract]))) OR ((("Endocrine System Diseases"[Mesh]) OR ("endocrine system disease*"[Title/Abstract])) OR ("endocrine disease*"[Title/Abstract]))) NOT (casereports[Filter]) AND (2012/1/1:3000/12/12[pdat])

**Topics and subtopics**

- Transition and referral
  - Definition of transition
  - Age of transition and care management
  - Transitional readiness
  - Patient referral to adult care
  - Multidisciplinary/interdisciplinary
  - Patient and caregiver education
- Sexual and bone development
  - Fertility, pregnancy, contraception
  - Bone health
  - HRT
  - Sexuality
  - Oncological risk
- Social and psychological aspects
  - Quality and style of life
  - Social barriers
  - Burden of chronic disease
  - Cognitive impairment
  - Eating disorders
- Systemic and metabolic disorders
  - Hypertension
  - Liver disease
  - Cardiovascular events
  - Autoimmunity
  - Diabetes
  - Overweight, obesity
  - Sensorial

**Working subgroups**

Subgroup A: Transition and referral

- Malgorzata Wasniewska
- Luisa de Sanctis (coordinator)
- Mariacarolina Salerno
- Maria Chiara Zatelli

Subgroup B: Sexual and bone health and oncological risk

- Maria Felicia Faienza (coordinator)
- Claudia Giavoli
- Maria Cristina Meriggiola
- Eriselda Profka

Subgroup C: Social and psychological aspects

- Tommaso Aversa (coordinator)
- Emanuela Scarano
- Stefano Stagi
- Roberta D’Aprile (psychologist)

Subgroup D: Systemic/metabolic disorders

- Alessandra Gambineri (coordinator)
- Anna Balducci
- Carolina Di Somma
- Armando Grossi
